# Supplementary material for: Interplay between MRI-based axon diameter and myelination estimates in macaque and human brain
Source: Imaging Neurosci (Camb). 2025 May 12;3:imag_a_00576. doi: 10.1162/imag_a_00576 (PMC12319738; doi:10.1162/imag_a_00576)
Supplement: Supplementary Material [file imag_a_00576-supp.pdf]

Supplementary materials

S1. T2 and SNR measurement in each sample

We estimated the voxel-wise T2 map by fitting the MSME data as generated from one T2 component (Björk et al., 2016), which showed the T2 in the human brain sample being much longer than the macaque brain samples (Figure S1). The macaque brain samples had overall similar T2 values with macaque 1 being the shortest and macaque 2 being the longest, which led to lower SNR level in macaque 1 while it was acquired with same protocol as macaque 2. Please see details in Table 1.

For SNR measurement of diffusion MRI data, we estimated the SNR maps for each sample with the preprocessed data as the ratio between the mean and standard deviation of the 8 b=0 images. The mean SNR levels of the diffusion MRI data were reported on WM voxels in Table 1. The WM voxels were defined by fractional anisotropy >=0.2 estimated from diffusion tensor model fitting.

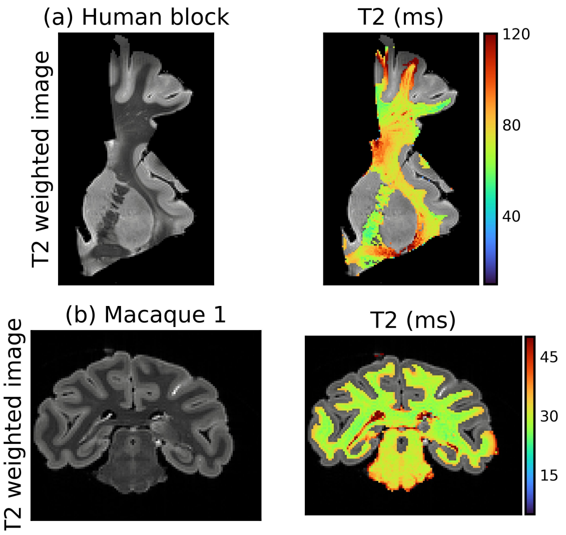

Figure S1. Example T2 maps from human and macaque samples.

S2. MSME signal decay and T2 spectrum

We estimated T2 spectrum from MSME data where the shorter T2 were considered as from myelin water and hence used for calculating MWF by setting a cut-off value. We used different T2 ranges to estimate the MWF in macaque and human samples based on their T2 spectrum. Below are spectrums estimated without regularization and with the ‘lcurve’ method for choosing the Tikhonov regularization parameters in the DECAES.jl package (v0.4.5) (Doucette et al., 2020). When estimating without regularization, the number of T2 values on the spectrum was set to the number of echo times in the acquired data; for regularized estimation, 100 T2 values were used.

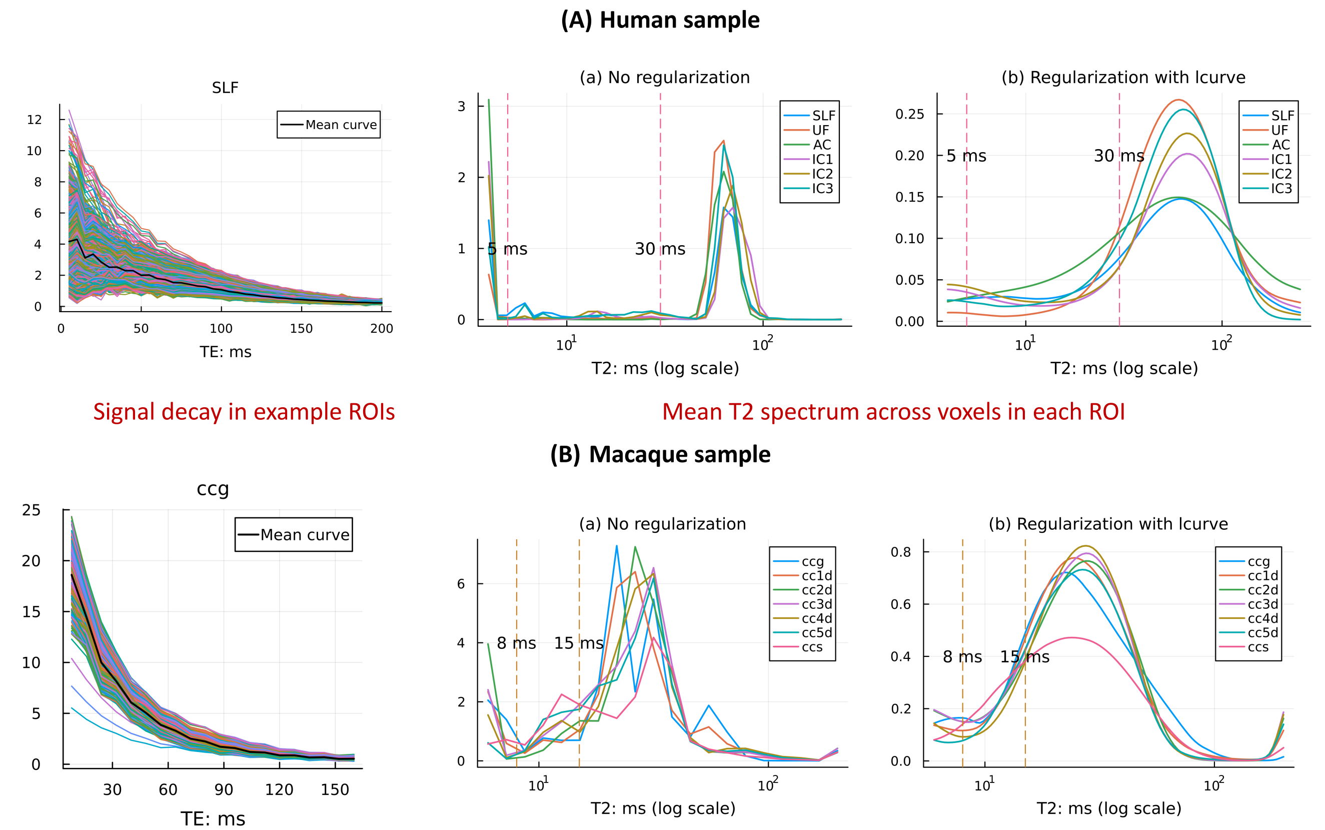

Figure S2. Signal decay and T2 spectrum from human(A) and macaque sample(B). For the signal decay curve, curves from all voxels within an ROI and the mean curve were given; for the T2 spectrum, the mean spectrums across voxels in each ROI were given**.**

S3. Inspection of diffusion MRI signal drift

We used the 8 interleaved b=0 images (in the beginning of each b-shell) to check if there was any signal drift during long scan hours (Vos et al., 2017) and found it to be negligible in all our samples. Below shows the mean signal intensities for all the measurements with error bars indicating the standard deviations of signals intensities for the b=0 measurements.

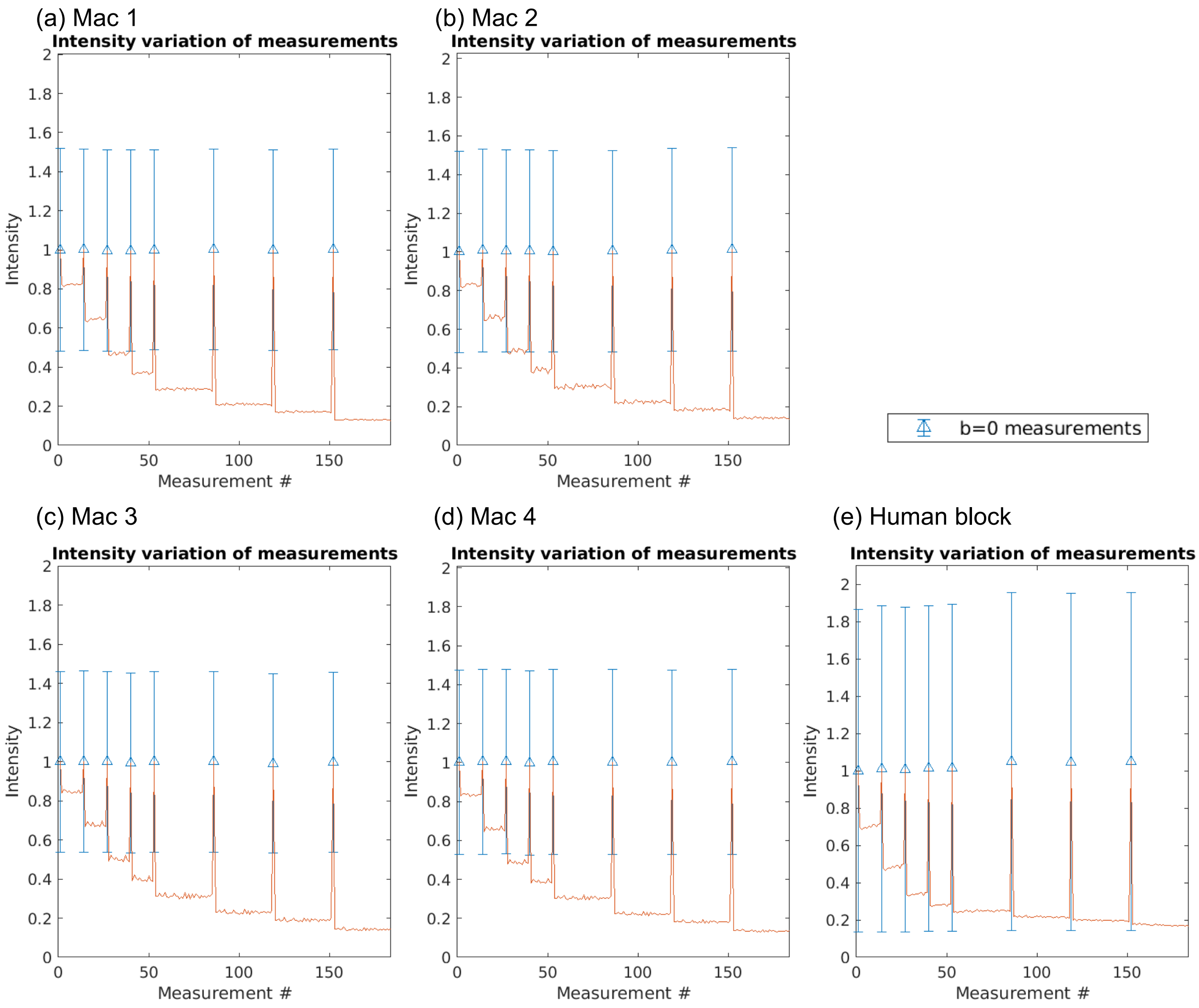

Figure S3. The mean signals across brain in all the measurements. The blue triangle markers indicate the b=0 measurements for each b-shell and the error bars indicate the standard deviations of these measurements.

S4. Evaluation of denoising methods

We saved complexed diffusion MRI and MSME data from macaque 2 for comparing the following denoising strategies.

a. Directly denoise the magnitude data with MPPCA (Veraart et al., 2016b, 2016a).
b. Denoising real-valued data. We retrieve the real-valued image data following the procedure described in (Eichner et al., 2015; Fan et al., 2020). The major implementation difference is that we apply 3D Fourier transformations and 3D Hanning windows as both our dMRI and MSME data are acquired using 3D imaging sequences. The imaginary part of the images containing purely noise is discarded while the real-

valued images are assumed to contain only additive Gaussian noise and we further denoise them using MCPCA.

c. Denoising complex data. We concatenate the real and imaginary parts of the complex images so that the data contains 2*N volumes (N is the number of DW measurements) and each volume contains Gaussian noise. We then estimate noise level and denoise the data using MPPCA. The denoised real and imaginary volumes are combined to magnitude data.

We compared the decay curve of denoised signals used for modelling. We use the direction-averaged signals verses b-values for diffusion MRI and the signals verses echo times for MSME data. Figure S1 shows the mean signal decay curves from small ROIs (9 voxels) in the splenium of corpus callosum. we didn't observe apparent differences of the decay curves from three denoising strategies. This suggests a relatively high SNR level in our ex vivo datasets compared to previous in vivo studies. Figure 2-3 further shows the denoised image maps from diffusion MRI and MSME. The difference between different denoising method can be visually seen from the background but not within the brain region.

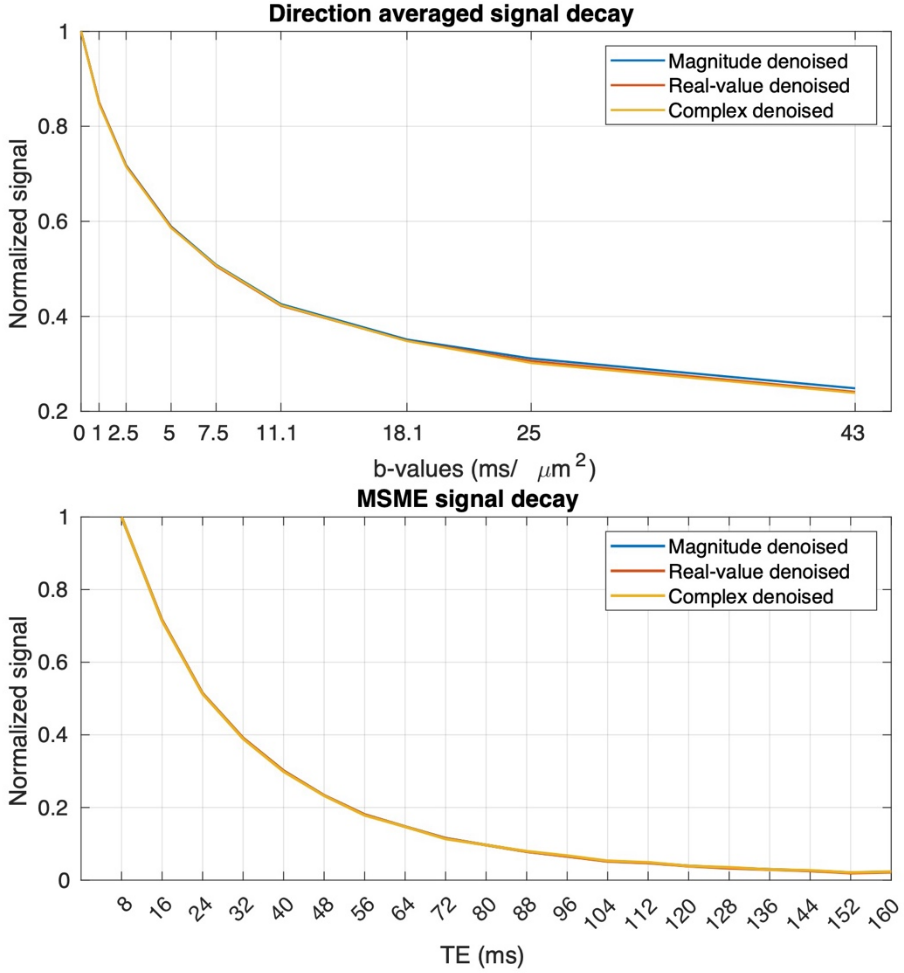

Figure S4.1. Signal decay curves of (a) direction-averaged dMRI signals and (b) MSME signals.

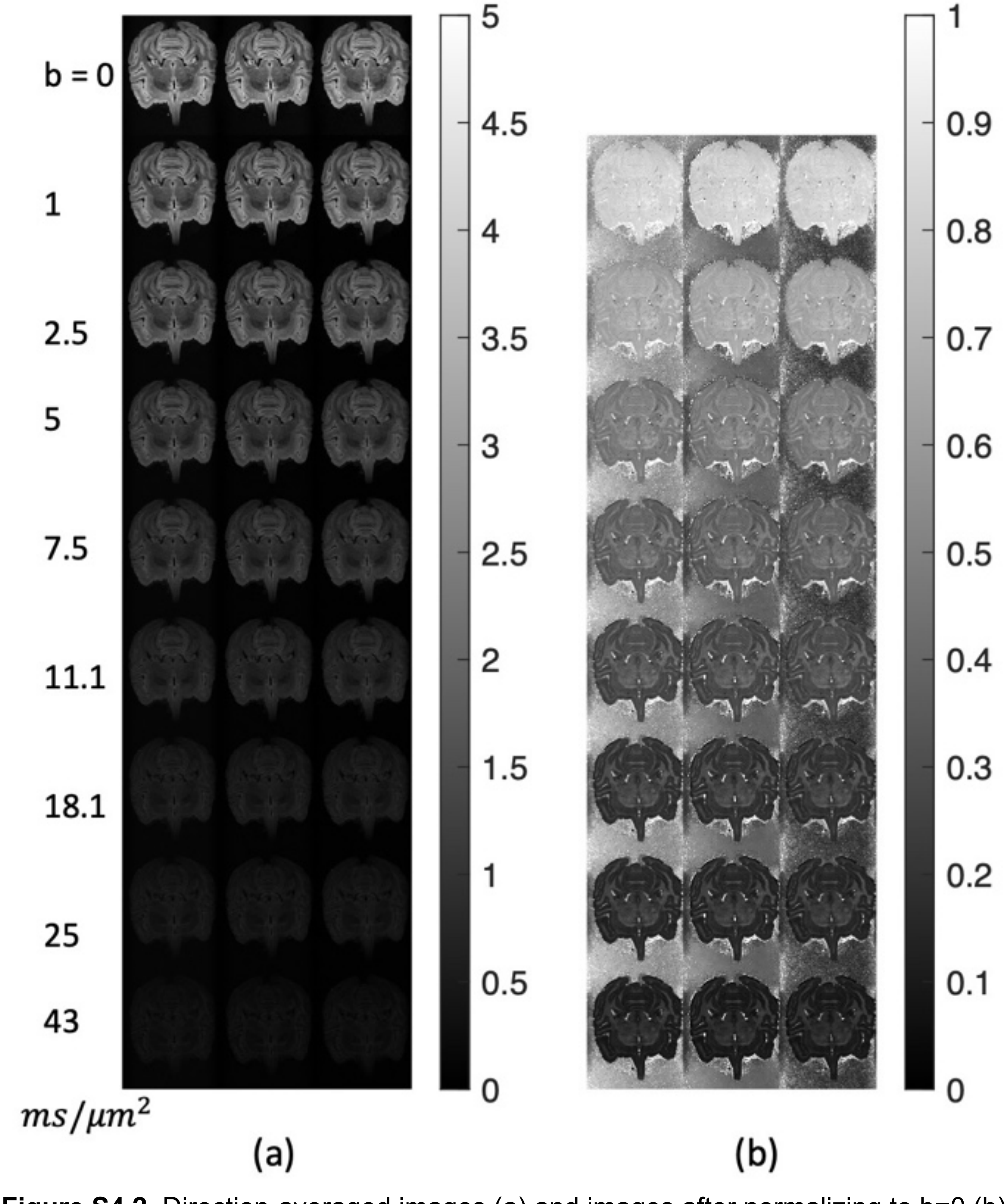

Figure S4.2. Direction-averaged images (a) and images after normalizing to b=0 (b). In (a) and (b), the first column shows magnitude denoised images, followed by real-value denoised and complex denoised images.

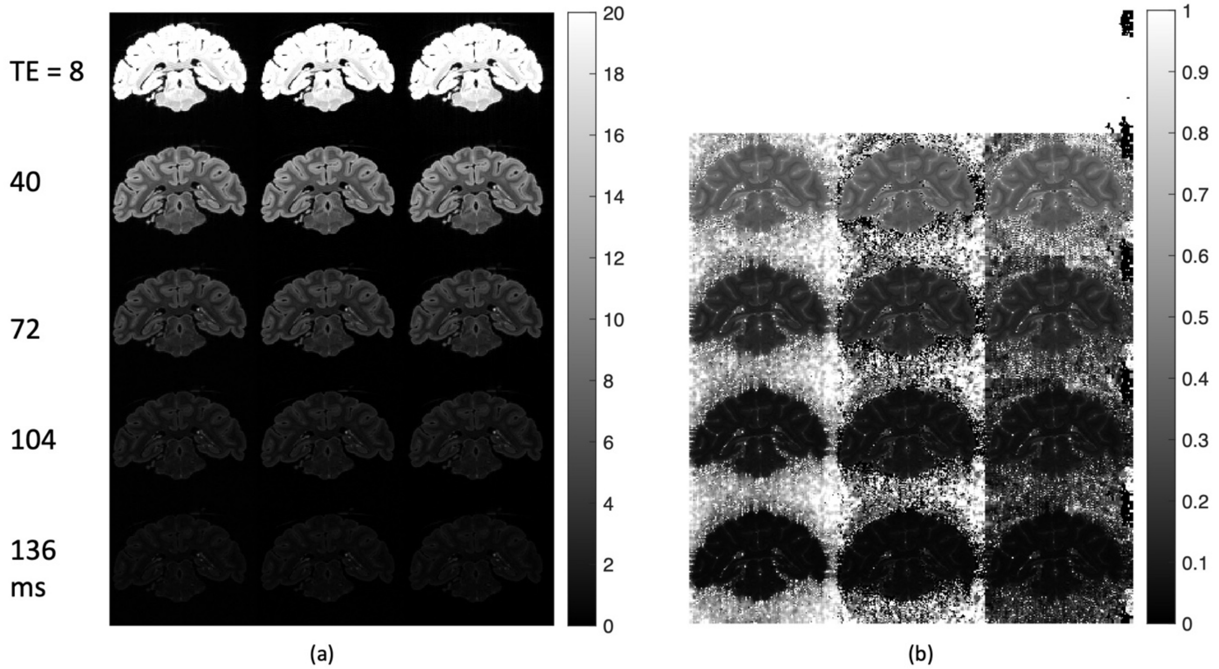

Figure S4.3. MSME images (a) and normalized MSME images (b) (normalized to images at shortest echo time (TE)). In (a) and (b), the first column shows magnitude denoised images, followed by real value denoised and complex denoised images.

S5. Inspecting dot signals in spherical mean and diffusion-weighted measurements

The dot compartment is observed as isotropically restricted and non-decaying signals. We inspected such signals in the spherical mean measurements and additionally in diffusion-weighted measurements before direction averaging in a single-fiber region to examine the directional dependence of the dot compartment. We chose a voxel with high FA in the corpus callosum in the macaque brain and the anterior commissure in the human brain.

We found that the dot signal estimated from spherical mean signal decay mostly represents the remaining high-b signals in the directions parallel to the fiber orientation. This agrees with a previous study (Veraart et al., 2020), which estimated the dot compartment signal fraction as the parallel signal in the corpus callosum with very high b-value using a separate scan and subtracted it from the data prior to axon diameter estimation.

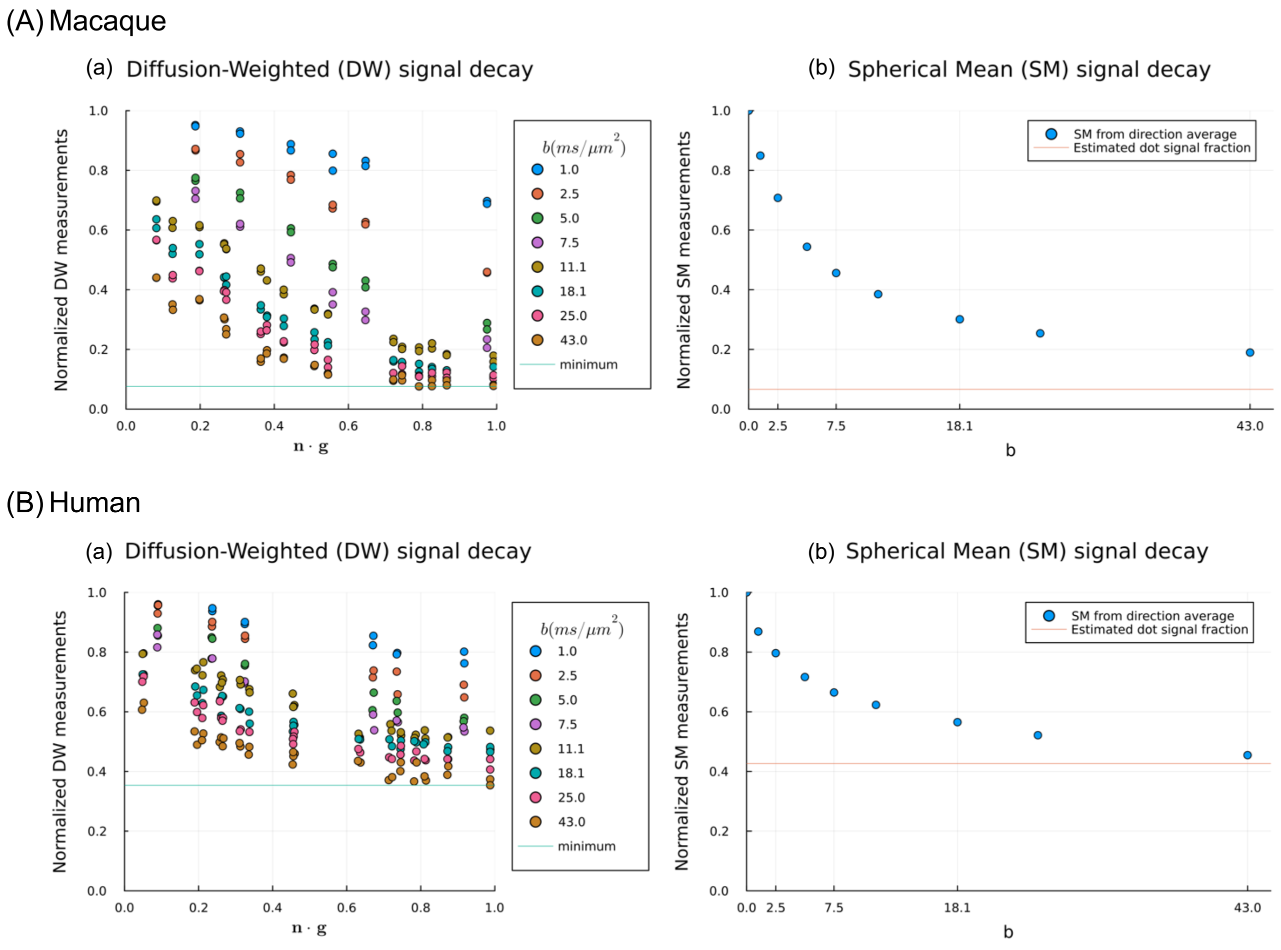

Figure S5. "Dot compartment" assessed in a voxel with high diffusion anisotropy in one of the (A) macaque and (B) human sample. **(a) Diffusion-weighted signal decay for different diffusion-encoding gradient directions.** The x-axis is the dot product between the major fiber direction **n** in the voxel and the diffusion gradient direction **g** (0: diffusion gradient perpendicular to the major fiber direction; 1: diffusion gradient parallel to the major fiber direction). **(b) Spherical mean signal decay.** The estimated dot signal fractions from the axon diameter model (horizontal line in (a)) were found to be very similar to the remaining signals at the highest b-value and in the gradient direction approximately parallel to the fiber orientation (horizontal line in (a)).

References

Björk, M., Zachariah, D., Kullberg, J., Stoica, P., 2016. A multicomponent T2 relaxometry algorithm for myelin water imaging of the brain. Magn Reson Med 75, 390–402. https://doi.org/10.1002/MRM.25583/ASSET/SUPINFO/MRM25583-SUP-0001-SUPPFIG1.PDF

Doucette, J., Kames, C., Rauscher, A., 2020. DECAES - DEcomposition and Component Analysis of Exponential Signals. Z Med Phys 30, 271–278. https://doi.org/10.1016/J.ZEMEDI.2020.04.001

Eichner, C., Cauley, S.F., Cohen-Adad, J., Möller, H.E., Turner, R., Setsompop, K., Wald, L.L., 2015. Real Diffusion-Weighted MRI Enabling True Signal Averaging and Increased Diffusion Contrast. Neuroimage 122, 373. https://doi.org/10.1016/J.NEUROIMAGE.2015.07.074

Fan, Q., Nummenmaa, A., Witzel, T., Ohringer, N., Tian, Q., Setsompop, K., Klawiter, E.C., Rosen, B.R., Wald, L.L., Huang, S.Y., 2020. Axon diameter index estimation independent of fiber orientation distribution using high-gradient diffusion MRI. Neuroimage 222. https://doi.org/10.1016/j.neuroimage.2020.117197

Veraart, J., Fieremans, E., Novikov, D.S., 2016a. Diffusion MRI noise mapping using random matrix theory. Magn Reson Med 76, 1582–1593. https://doi.org/10.1002/mrm.26059

Veraart, J., Novikov, D.S., Christiaens, D., Ades-aron, B., Sijbers, J., Fieremans, E., 2016b. Denoising of diffusion MRI using random matrix theory. Neuroimage 142. https://doi.org/10.1016/j.neuroimage.2016.08.016

Veraart, J., Nunes, D., Rudrapatna, U., Fieremans, E., Jones, D.K., Novikov, D.S., Shemesh, N., 2020. Noninvasive quantification of axon radii using diffusion MRI. Elife 9. https://doi.org/10.7554/eLife.49855

Vos, S.B., Tax, C.M.W., Luijten, P.R., Ourselin, S., Leemans, A., Froeling, M., 2017. The importance of correcting for signal drift in diffusion MRI. Magn Reson Med 77. https://doi.org/10.1002/mrm.26124
